# Doctors’ experiences regarding patient referral to tertiary care in Pretoria, South Africa

**DOI:** 10.4102/jcmsa.v2i1.46

**Published:** 2024-01-29

**Authors:** Maureen N. Mothupi, John V. Ndimande, M. Adeline Nkoane, Carian Steyn, Tombo Bongongo

**Affiliations:** 1 Department of Family Medicine and Primary Health Care, School of Medicine, Sefako Makgatho Health Sciences University, Pretoria, South Africa

**Keywords:** experiences, referring doctors, patient referral, Pretoria, South Africa

## Abstract

**Background:**

Patient referral is an essential component of patient care that cannot be underscored. Despite the fact that the regulations and steps involved are well known, little is known about the experiences of referring doctors during the process, as well as the factors that support or impede the referral. This study aimed to investigate doctor’s experiences regarding patient referral to tertiary care in Pretoria, South Africa.

**Methods:**

A qualitative study using a descriptive phenomenology design and involving one-on-one interviews with eight doctors from Jubilee District Hospital who had referred patients to Dr George Mukhari Academic Hospital, in Pretoria, South Africa.

**Results:**

Eight themes were identified from the analysis: estimated time taken to refer patient, attitudes of the receiving doctors towards patient referral, applicability of the receiving doctors’ advice prior to the referral, referring doctors’ perceptions regarding the receiving doctors’ attitudes towards patient referral, consequences of delays on patient care; strategies to address referral issues, experience regarding transportation of patients being referred and recommendations to improve the referral system. These are further expanded upon by six related sub-themes.

**Conclusion:**

The referral process from a district hospital (Jubilee Hospital) to a tertiary care (Dr George Mukhari Academic Hospital) is onerous, with barriers being experienced at almost every step. A variety of issues hindered this process, including administrative, logistical and communication barriers that left referring doctors almost demotivated.

**Contribution:**

The challenging experiences from referral highlight the significance of paying close attention to patient referral in order to avoid compromising patient care.

## Introduction

Patient referral is the transfer of a patient from an institution with limited resources to an institution with more resources or more specialised. This involves a number of steps that have to be taken by the doctor who is transferring the patient.^1,2^ It is also the mechanisms through which doctors and institutions interact and collaborate in order to maintain, enhance and restore an individual’s health.^2^ A patient’s referral to another level of care might be performed internally, upwards, downwards or laterally when it happens within health institutions of the same level of care for continuity of care.^2^

More than 60 000 medical records of patients who visited community health centres (CHCs) in the Western Cape province of South Africa in 2006 revealed that 20% of patients were referred to hospitals for ultrasound, more than 15% for incision and draining (I & D), and 13% for other reasons; 20% of the referrals from the CHCs to hospitals were deemed unnecessary.^3^ Patient referral is influenced by various factors, including the duration required to transfer a patient from one facility to another, the perceptions of referring doctors regarding the willingness of receiving physicians to accept referrals, recommendations provided to referring doctors by the receiving doctors for stabilising patients before referral and the experience with the transportation process for referred patients.

In another study carried out in the Western Cape province of South Africa, some referring doctors perceived that there was poor communication from the receiving specialists at tertiary care hospitals.^4^ From this survey, receiving specialists would communicate better if they received a referral letter, if they knew the referring doctor and if they recognised the coordinating role of primary care.

When a patient arrives at a hospital with a medical concern that falls beyond the expertise of the attending doctor, it becomes necessary to transfer the patient to a specialised level of care.^5^ Communication between the referring and receiving hospitals is, however, paramount to ensure optimal care. Before every patient is transported, the ABCD of preparation should be followed. Another important aspect to take into consideration is the correct mode of transport, either by ambulance or by helicopter for example.^5^ The mode of transport may affect the patient’s physiology, and therefore sending doctors should be well advised on how to best prepare a patient for transport depending on the mode that will be used.^5^ Important to notice is that the correct level of emergency personnel should be arranged prior to the transfer the type of patients participating.

In Johannesburg, it was observed that children decompensate more quickly than adults, which underscored the need for performing such a study. When a facility is unable to give the requisite level of decisive care, patients must be referred and transported to alternative facilities that are better suited to provide optimum treatment and care. Communication channels and a cohesive team approach involving all role players from receiving and referring facilities, as well as emergency medical services (EMS) call centres and pre-hospital care providers, should be established. The participants’ experiences in this study show that there is still much work to be performed in this area within the South African public healthcare system.^6^ Healthcare administrators must take steps to ensure that their systems for transferring ill children have minimal time delays and well-defined communication paths between the dispatching and receiving facilities.

In turn, EMS managers need to ensure that transfers are conducted by properly qualified ambulance crews who are equipped with the appropriate resources.^6^ In South Africa, ambulance services must ensure the safety of the person transported, carry out regulated procedures of patient transfer between hospitals or health institutions and dispatch ambulances according to stipulated standards, as guided by the *South African National Health Care Act* of 2021.^7^

Between January 2022 and February 2022, out of 2478 patients examined in the casualty department at Jubilee District Hospital (JDH), 64 (2.58%), patients were sent to Dr George Mukhari Academic Hospital (DGMAH) (tertiary level of care) in Pretoria for further management, as shown by the casualty register. Jubilee District Hospital is a classified district hospital and is actually the bridge between primary and tertiary care; it covers 32 clinics. Because of a limited number of employees and limited resources compared to the size of the catchment population and the hospital’s scope of practice, the district hospital regularly struggles to keep up with the workload. As a result, some patients will not be managed at this level, they will be referred to DGMAH (next level of care) for further care. Because of the number of referrals as well as the time that can take one referral, this study explored the JDH doctors’ experiences regarding patient referral to tertiary care in Pretoria, South Africa.

## Research methods

### Study design

This was a qualitative study using a descriptive phenomenology design, as experiences of a phenomenon known as ‘patient referral’ were explored. Eight one-on-one interviews were conducted.

### Setting

The study was carried out at JDH, which is located in Hammanskraal, a rural area near Pretoria on the far Northern boundary of the Gauteng province of South Africa. It has 551 beds and covers 32 clinics, of which 11 are in Gauteng province and 21 are in the North West province of South Africa. It provides paediatrics, obstetrics and gynaecology services, internal medicine, general surgery, family medicine and anaesthesia, among other medical services.

### Study population, sampling and sample size

All 44 doctors employed by JDH were included in the study population and a purposive sampling technique was adopted. The sample size of this study was eight (*n* = 8) respondents, because of the availability of respondents and their agreeing to take part in the study, as well as the attainment of data saturation.

### Data collection

The principal researcher (PR) notified all doctors about the study, the day, time and venue of the interviews. The communication was verbal and via the hospital’s WhatsApp group. A few hours prior to the interviews, each medical doctor received a call on his or her personal phone. At the venue, the PR greeted the respondents and provided an overview of the agenda. A research assistant (RA) was introduced to the respondents. The RA was a retired psychiatric nurse, experienced in quality data collection, trained by the PR on how to present the research study’s topic as well as its goal and objectives to the respondents. All the respondents signed the consent forms and were given the option to withdraw should they feel uncomfortable during the interview. It was stated that English was to be the language of conversation and that no remuneration was attached to participation in the study. Principal researchers iPhone was used to record the interviews, and Google Drive was used to save the audio files. One-on-one interviews were scheduled, and each respondent arrived at his or her appointed time.

Each respondent went through a semi-structured interview with six open-ended questions. The RA, who led the interviews, followed the interview guide’s six exploratory questions, which were as follows:

- Can you tell me about the time it takes for you to refer a patient to DGMAH?
- Can you tell me how receptive the receiving doctors are?
- How applicable is the procedural advice given to you by the receiving doctor, which you need to carry out before the patient is deemed stable enough for referral?
- Can you tell me about your experiences regarding the transportation of patients being referred?
- Can you tell me about any strategies or platforms in place to address the issue?
- Could you please tell me about the effects of referral delays on patient care?

### Data analysis

Data were transcribed and converted from the audio data to written text using Microsoft Word by a trained RA. The typed transcripts were cleaned and sent to the researcher to verify accuracy. The cleaned transcripts were then uploaded into NVivo 12 for analysis. Analysis of the transcripts was performed by identification of themes that were directly related to the research questions that detailed the experiences of the doctors during patient referrals. The interpretative philosophy was used to determine meaning from the responses given, and the data were grouped according to the objectives of the research study, making analysis context bound.

### Ethical considerations

Ethical clearance to conduct this study was obtained from the Sefako Makgatho Health Sciences University Research Ethics Committee (SMUREC) (No. SMUREC/M/210/2018). JDH granted permission for the study. Confidentiality and anonymity were maintained throughout the research process. Participants gave informed consent and were informed that they could opt out at any time.

## Results

Eight respondents participated, comprising one paediatrician, one family physician, four medical officers and two community service doctors. The doctors worked in different departments within JDH, for example, Casualty, Internal Medicine and Paediatrics. The age of the participants ranged from 31 to 50 years, with five females and three males. The eight doctors participated in the semi-structured interviews. Due to the small number of participants no identifiers have been provided to maintain anonymity. A detailed narrative analysis of the themes and sub-themes that emerged is provided next (see Table 1).

**TABLE 1:** Themes and sub-themes.

No.	Themes	Sub-themes
1.	Estimated time taken to refer a patient from one healthcare facility to another	-
2.	Attitude of the receiving doctors towards patient referral	-
3.	Applicability of procedural advice given by receiving doctor	-
4.	Referral doctor’s perceptions regarding the reasons why receiving doctors refuse and/or reluctant to receive patients	-
5.	Consequences of referral delays on patient care	-
6.	Strategies to address referral issues	6.1 Third-party involvement 6.2 Doctor’s own efforts 6.3 Forums to address issues
7.	Experience regarding transportation of patients being referred	7.1 Delay issues 7.2 Forum for emergency medical services (EMS) complaints 7.3 Effectiveness of strategies
8.	Recommendations to improve referral system	-

### Theme 1: Time estimate for referring a patient from Jubilee District Hospital to Dr George Mukhari Academic Hospital

The required time from the decision to refer the patient to the point at which the patient was accepted for reference is considered, this takes more than 30 min. One participant said: ‘It can take about an hour or even more trying to refer one patient’.

### Theme 2: Attitude of the receiving doctors towards patient referral

This theme aimed at exploring the perceptions of the referring doctors regarding the attitudes of the receiving doctors regarding patient referral. There was a general perception of referrals being undesirable, and an unwillingness to receive patients: ‘But yeah, they’re not receptive to be honest’, said one participant; ‘Generally, they are not willing to receive patients’, said another participant.

### Theme 3: Prior to patient referral, the Dr George Mukhari Academic Hospital (receiving) doctor gave the Jubilee District Hospital (referring) doctor procedural instructions

Most of the doctors at JDH found the advice given by the receiving doctors as practical and applicable, for example, one participant mentioned: ‘Ok, most of the advises are very applicable; I can do them’; and another one said: ‘Most of them are practical’. However, other doctors argued that receiving doctors tell them to repeat procedures that they have already performed. Others saw these advises as stalling tactics in order to postpone receiving the patient: ‘Hmm, most of the time, they tell you something that you have already done’, declared one participant.

### Theme 4: Referring doctors’ perceptions regarding the reasons why Dr George Mukhari Academic Hospital (receiving) doctors refuse and/or are reluctant to receive patients

Issues pertaining to high volumes of patients that lead to increased workload and being overwhelmed were brought forth, while one participant raised the issue of erroneous referrals that contribute to reluctance to accept patients: ‘Another thing, probably because of the workload that side, at times you find that they are quite reluctant to take patients’, said one of the participants.

### Theme 5: Consequences of referral delays on patient care

This theme indicated a general concern about the effects of referral barriers on patients. There were noticeable repercussions of the referral delays, and most participants viewed refusal or delaying referral as a violation of patient rights, as they are being denied access to healthcare: ‘It’s sad because it’s delaying patient care. Yeah, it’s actually delay – in fact, I would say we are denying our patients their basic rights. Yeah, so it’s not necessary’, as stated by one participant. From another participant:

‘Remember that patient is not the only patient that is waiting for you. There are others who are also waiting for you. And dealing with this one patient for one hour when you have 30–40 more patients waiting for you, will also delay others.’

### Theme 6: Strategies to address referral issues

It was observed that in the light of so many issues surrounding patient referrals, various strategies have been implemented to address and assist in the process.

#### Sub-theme 6.1 Third-party involvement

One strategy was the involvement of a third party as a mediator between the referring doctor and receiving doctor. In instances where doctors who were referring encountered problems, they had the option of talking to a consultant on call at DGMAH. One participant described the role of the consultant as ‘To help us when we are stuck’ and further indicated:

‘When I have a case at night, and I don’t know what to do, I think that’s where I can pick up my phone and call a consultant because I would have done 1, 2, 3, 4 and followed the protocol like I’m supposed to and I’m still not getting it right. Then I’ll say, OK let me call my consultant. Also, if I’m in doubt with what to do, I will call the consultant on call at DGMAH.’

Other participants indicated that they involved clinical managers or Heads of Department when they encountered issues with receiving doctors. Most of the doctors had a positive experience when their issues were escalated to a third party, as detailed: ‘For example, the registrar might call you saying “I don’t have a bed, wait for me. Let’s create a bed first” and then you call in the morning’, at that time the answer might be: ‘No, we are still in the post intake round. Wait for us to do rounds and see discharges’.

‘No, we are still in the post intake round. Wait for us to do rounds and see discharges’.

But once you talk to the consultant, and the consultant says send the patient, then suddenly, the bed is there, as one of the participants stated. Another participant agreed with this statement, saying:

‘I won’t just leave the patient like that. If I must call the consultant of the department, I will do that. You can use the clinical manager this side and then they call the MMO (Managing Medical Officer who is the back up for the entire city) to say we have got such a patient and the doctor or registrar that side is not assisting.’

It seems the bed appears to become available once the case has been escalated.

#### Sub-theme 6.2: Doctors’ own efforts

In some cases, doctors had to resort to their own efforts to overcome referral issues from receiving doctors, such as transferring patients to an alternative hospital (such as Odi District Hospital, another district hospital that refers to DGMAH) or being forceful and persistent to ensure that the receiving doctor accepts the patient. For example, when asked what she would do if the referral process is prolonged, one participant responded as follows:

‘I’m very pushy, so I give you 15 minutes, it’s too much to discuss what I have discussed with you with the consultant. You can discuss with the consultant and come up with a decision, then come back to me.’

The same participant added:

‘I don’t like to wait for an hour without a response. So, half the time I will call back to remind the colleague: “You said you will come back to me after talking to the consultant and since you are answering my call, it means you are no longer on the call with the consultant.”’

#### Sub-theme 6.3: Forums to address issues

Evidently, there was a platform where doctors could discuss the barriers they encounter when referring a patient. As one of the doctors indicated: ‘There’s a forum that’s a cluster meeting that’s where our challenges are taken to’. This was confirmed by his colleagues who added, ‘Yeah, we do that, we have got every Monday, Friday meetings. Yeah, clinical meetings. So, we raise individual incidences to our clinical manager’, confirmed another participant. However, these meetings were not effective as most doctors specified that things remain unchanged as summarised by this statement:

‘I feel discouraged. Sometimes you feel like these meetings are just there. But they’re not solving anything like the things that we have discussed the previous meeting. They will be just read as previous meeting minutes, but there will be no solutions’ [*stated another participant*].

### Theme 7: Experience regarding transportation of patients being referred

This theme has been divided into three sub-themes that describe the experiences of doctors regarding transportation of patients. This was described as a demanding process characterised by many delays. Interestingly, one participant mentioned that the role of organising transport was an unnecessary task on the part of doctors and could be administered by clerks:

‘Another issue is that in Jubilee District Hospital, unlike in other hospitals in the world, it is the doctor’s duty to arrange EMS (Emergency Medical Services) services for patients going to DGMH, which wastes vital time where we could be reviewing results, X-rays or even seeing other patients. I feel this can be handled by somebody else, even a clerk can organise transport you know.’

Additionally, participants felt that their complaints did not yield any positive outcomes, as strategies to resolve issues were not fruitful:

‘Yoh, Yoh! No, no, no. It’s a nightmare. But with the EMS, you will wait even though you mention to them “Listen, it’s an emergency and this patient cannot wait for longer than an hour”. I don’t know if they try, but still, you will wait. So, experiences with the EMS from my side, it’s not good’ [*declared one of the participants*].

#### Sub-theme 7.1: Delay issues

One the main issues contributing towards delays was that the EMS firstly had to confirm with the receiving doctor before transferring the patient, as per their protocol. The participants mentioned how cumbersome this is, as it further delays the process. In the event that EMS is unable to contact the doctor, then the patient will be brought back to JDH (the referral hospital). The following concerns were voiced by the participants:

‘Yes, they called the hospital. They said that I need to verify the transfer with the receiving doctor. If EMS cannot get through to the receiving doctor’s number, they would not take the patient because now we find that if we are trying to refer a patient to a surgeon and the surgeon has accepted the patient but now is busy in theatre operating on another patient, he’s not in a position to answer his phone, and this means that now the patient will be stuck with you here in Jubilee hospital until such time that they manage to get through to the doctor’s cell and speak to him’ [*said one participant*].

Communication barriers refer to the various hindrances that led to poor communication or delayed responses and were one of the issues that doctors often complained about that result in delays when patients have to be transferred from JDH:

‘There are instances when you’ll be able to reach the switchboard operators, although it may be after a lengthy phone call. They will give us more than one telephonic extension. Which at the end seem not going through’ [*as stated one of the participants*].

The participants further mentioned that transport will take a long time to come, pointing out issues related to being short-staffed as well as the lack of resources:

‘But most of the time, you have to wait and wait. You can make at least three phone calls to follow up that you’re still waiting. It’s been 30 minutes or an hour and there is no one. They will tell you the advanced paramedic went to collect a patient somewhere else and as soon as they come back, you will be next on the list’ [*as explained one participant*].

It was further added that:

‘Several times you find that you call, or arrange transfer with the physician, you book transfer with EMS, they give you a reference number and it will take forever for them to come. You arrange a transfer around 11 mid-day and the ambulance can even come after 7 pm’ [*as complained one of the participants*].

Another participant supported the experiences detailed by the co-workers and added that:

‘You wait for a bit longer because the ambulance is somewhere in Johannesburg (neighbouring city) so they have to get the ambulance in Pretoria in order to get the patient over to that side.’

It was perceived by the participants that the EMS paramedics do not recognise urgency and can fail to act effectively. The following statement further elaborates on their frustrations: ‘I don’t think they have understood that there is an emergency, since they are taking long to come and collect the patient’.

It was mentioned that there are different transportation requirements for different patients, as the condition of the patient will determine the specialised care that is required. Therefore, it is a prerequisite that when doctors arrange for transportation services, they should provide a detailed explanation of the condition of the patient that needs to be transferred. However, there were complaints that despite the doctors providing detailed information about the patient; in some cases, EMS sends paramedics who are not skilled in transferring patients, and they often have to go back and dispatch the right paramedics, which results in another delay. This left doctor frustrated, as one participant complained:

‘Sometimes when you call, you have to let the paramedics know what kind of patient you are referring. And they have to send their relevant people with their relevant expertise or experience for that particular condition of the patient that you are referring. Sometimes they just send people who do not have the scope of handling what you have referred and the people who come here say “No, we can’t take this because the patient has a drip or we don’t take patients with drips”, things like that, and you ask yourself but why did you come? Because I called and I said the patient is having this condition and they should have brought somebody who can deal with a patient with a drip. So, if you’re coming here, and if you say you can’t take this patient because the patient has a drip, then it’s a problem for me because initially when I called, they should have sent somebody who has experience.’

Others complained about EMS not bringing the required resources for the type of patient that is to be received:

‘You booked a patient before somewhere, so when you booked, they ask you is there any attachment, you tell them the patient is on oxygen. But surprisingly, the paramedic comes with no wheelchair, with nothing. Then once they check, some will say: “No, this one need advanced paramedic” or they will come and take her away.’

#### Sub-theme 7.2 Forum for Emergency Medical Services complaints

This theme emerged from a follow-up question that asked whether there was a platform for doctors to voice their frustrations about the transportation issues that they face when they refer patients. It was observed that doctors are provided with such a platform as the management of the hospital has meetings with the EMS management team to air grievances faced. The following provides a glimpse of how doctors responded:

‘Our clinical manager and some of the Head of Department last year; I can’t remember the month but towards the end of the year, they communicated, or they had a meeting with our founding EMS and certain grievances were discussed with them.’

#### Sub-theme 7.3 Effectiveness of strategies

One of the strategies that were implemented was the introduction of an administration book in the Casualty Department that indicates when the doctors called for EMS as well as the time of arrival of EMS. This was aimed at providing evidence and keeping a record of arrival times to improve efficiency and accountability. One participant stated:

‘So now what we have done now in casualty, we put a book, time that you call for EMS, time that they came and responded and the time that they picked up the patient.’

This was supported by another participant who mentioned:

‘We have a book in casualty that you indicate when you called the EMS when the EMS arrived. To me that book is just a waste of time. It’s a waste of resources because the problem is still there. So, but I guess maybe it’s been evidence that they can take to the EMS and say, okay, so this this is what we have.’

When asked about how effective the strategies are, one participant said:

‘Now, we are going to look at that data and if we have a specific remark, we put it there and then when we are in that meeting, we show to them “This is what is happening”. And we can say it’s getting a little bit better.’

### Theme 8: Recommendations to improve the referral system

Various recommendations were suggested by the doctors to improve the overall referral procedure. The need for support was one of the main issues that was voiced out, as it was said, ‘We need support, yes. We need support, we also need to be considerate’. It was also perceived that receiving doctors do not have knowledge about the challenges faced in JDH. One participant complained:

‘I think it’s that they don’t know the reality of Jubilee and it would be nice if those registrars at DGMAH, they shouldn’t just be trained in the tertiary hospitals, they should know what is happening in the surroundings. They should have a strong outreach system so that when a doctor calls you from Jubilee hospital, you understand what this doctor is facing.’

Thus, it was suggested by the same doctor that a strong outreach system is implemented so that receiving doctors are knowledgeable about the conditions, such as the lack of resources within JDH. Other suggestions that were provided are as follows:

‘If they can build this hospital to have enough resources to become a secondary level, then we have a district hospital within this community, and we put doctors in the clinics, maybe we will have patients who are not coming late to the hospital. Maybe we will get people who are seeking help on time. We are talking about prevention and family medicine and now it’s just a curative model. The bottleneck has to be removed. That’s my point.’

Said one participant. Some recommendations were made such as:

‘First recommendation would be that call roster should be made available on daily basis so that we know who the receiving doctors are. Number 2, many doctors in DGMH especially registrars are people who are there to learn, should be readily receptive to patients, either in the form of accepting patients or discussing and treating the patient over the phone through what you are telling them’ [*as said one participant*].

‘And follow up from their side, the transfer back letters should be made clear’, added the same participant.

‘Drs need to write a transfer back letter stating that this is what we found, and this is what we did, and this is what we expect. If me as the transferring doctor, I did something wrong, they must also include that and tell me that “Dr or colleagues, this is what we found, you missed an opportunity”’ [*as mentioned another participant*].

‘Medicine requires continuous learning; it’s part of the principle and we must teach each other. Yes, I saw your patient and that was it, this was or it was not that. Learn to be humble to each and teach each other in a respectful manner, because sometimes you will find other people who will belittle you, and for some of us it forces us to act in an adult manner’ [*as concluded one participant*].

## Discussion

As respondents expressed themselves, nearly all of them in this study appeared discontented with patient referral from a district hospital to a tertiary care level. A qualitative study conducted in Norway had similar results, with general practitioners (GPs) being blamed by receiving doctors (specialists) for this patient referral failure. It was claimed that using good referrals would have sped up the referral process. General practitioners were accused of referring instances that were unnecessary.^8^ In the eyes of Norway GPs as well as Jubilee respondents, such an argument may be perceived as a means of denying or delaying patient referral.

The time that it takes to transfer a patient from one facility (JDH) to another (DGMAH) was seen to be delayed. According to the respondents, the time taken for a doctor to arrange a referral with the receiving doctor is longer than 30 min. A similar delay in referral time was observed in a Rwandan study, where transporting a patient from one hospital to another can take up to 2 h.^9^ According to the Jubilee respondents, the delay might be caused by a variety of circumstances, including the transportation challenges and a lack of referral time for patient referral under the South African Referral Policy on Health Services.^2^ An additional reason on the refusal or delay was patient too sick to be admitted according to a KwaZulu-Natal study (province of South Africa).^10^ Perhaps the outcome in a tertiary level of care may not differ from the district level.

When it comes to transporting patients from JDH to DGMAH, the process has been described as challenging and hampered by multiple delays. This can include a delay when phoning the EMS control room because of a busy line, a shortage of ambulances, a need to confirm with the receiving doctor before transporting the patient to him or her and other issues. This is consistent with the results of a study conducted in Ethiopia,^11^ which showed that the time required to transport a patient to the receiving hospital was two times longer than the advised amount of time, which is less than 8 min.^11^ However, unlike this investigation, the Ethiopian study did not identify the primary cause of the delay and concluded that there is a shortage of highly trained emergency personnel who can assist in the transportation of critically ill patients.^11^ The Ethiopian’s finding seems to be consistent with what was raised in a study of the experiences of emergency care providers conducting critical care transfer in South Africa, which stated that the basic EMS syllabus is inadequate in preparing the emergency personnel to handle emergencies fully.^12^ The EMS lacks the necessary human resources for handling patients who need specialised equipment such as ventilator machines. A study in KwaZulu-Natal in South Africa stipulated^13^ that the advanced life support personnel are leaving the country to work overseas because of unsatisfactory job conditions coupled with financial considerations. Plans to retain them within the country are failing^13^ – hence the lack of resources that also contributes to the delay that affects the patient referrals, therefore to patient care.

## Conclusion

The patient referral process from Jubilee district hospital (JDH) to tertiary care (DGMAH) in Pretoria, South Africa, is onerous; with barriers being experienced at almost every step. There were a variety of issues hindering this process, ranging from administrative to logistical and communication barriers, that left referring doctors almost demotivated. There is a need to emphasise the two major issues that come out of the research, which are the unwillingness of the receiving doctors and the difficulties with EMS. Strategies to remedy the issue were ineffective.

### Recommendation

As patient referral is an integral part of patient care, it is recommended that more studies regarding patient referral and factors helping and hindering referrals should be carried out, with more emphasis on the interaction or communication process between the referring and receiving doctors.

### Limitations

There are limitations when it comes to the applicability of this study’s findings to the entire city, as only eight participants agreed to take part in the study out of a total of 44 doctors employed at the facility who were contacted, and only one hospital as a site was considered.
